# Moderation effect of mammography screening among women with multiple chronic conditions

**DOI:** 10.1038/s41598-022-06187-7

**Published:** 2022-02-10

**Authors:** Hui-Min Hsieh, Cheng-Ting Shen, Ling-Sui Chen, Fang-Ming Chen, Shu-Chuan Yeh

**Affiliations:** 1 Department of Public Health, Kaohsiung Medical University, Kaohsiung, Taiwan, ROC; 2 Department of Medical Research, Kaohsiung Medical University Hospital, Kaohsiung, Taiwan, ROC; 3 Department of Community Medicine, Kaohsiung Medical University Hospital, Kaohsiung, Taiwan, ROC; 4 Center for Big Data Research, Kaohsiung Medical University, Kaohsiung, Taiwan, ROC; 5 Department of Family Medicine, Kaohsiung Municipal Ta-Tung Hospital, Kaohsiung, Taiwan, ROC; 6 Administration Center, Kaohsiung Medical University Hospital, Kaohsiung Medical University, Kaohsiung, Taiwan, ROC; 7 Department of Business Management: Doctoral Program, National Sun Yat-Sen University, Kaohsiung, Taiwan, ROC; 8 Department of Surgery, Kaohsiung Municipal Ta-Tung Hospital, Kaohsiung, Taiwan, ROC; 9 Division of Breast Oncology and Surgery, Kaohsiung Medical University Hospital, Kaohsiung, Taiwan, ROC; 10 Research Center for Environmental Medicine, Kaohsiung Medical University, Kaohsiung, Taiwan, ROC

**Keywords:** Medical research, Outcomes research, Breast cancer, Cancer epidemiology, Cancer screening, Breast cancer

## Abstract

Comorbidity substantially affects breast cancer risk and prognosis. However, women with chronic conditions are less likely to participate in mammography screening. Few studies have examined potential benefits of mammography in women with chronic conditions. This study investigated the moderation effects of mammography screening on early stage breast cancer and all-cause mortality among women aged 50–69 years with chronic conditions in Taiwan. We used a matched cohort design with four nationwide population databases, and an exact matching approach to match groups with different chronic conditions. Women population aged 50–69 years in 2010 in Taiwan were studied. A generic Charlson comorbidity index (CCI) measure was used to identify chronic illness burden. The sample sizes of each paired matched group with CCI scores of 0, 1, 2, or 3+ were 170,979 using a 1-to-1 exact matching. Conditional logistic regressions with interaction terms were used to test moderation effect, and adjusted predicted probabilities and marginal effects to quantify average and incremental chronic conditions associated with outcome measures. Statistical analyses were conducted in 2020–2021. Women with more chronic conditions were less likely to participate in mammography screening or to receive early breast cancer diagnoses, but were at greater risk of mortality. However, mammography participation increased the likelihood of early breast cancer diagnosis (OR 1.48, 95% CI 1.36–1.60) and decreased risk of all-cause mortality (HR 0.53, 95% CI 0.51–0.55). The interaction terms of CCI and mammography participation indicated significantly increased benefits of early breast cancer diagnosis and decreased risk of all-cause mortality as chronic illness increased. Mammography participation significantly moderated the link between comorbidity and outcome measures among women with chronic conditions. Hence, it is important for public health policy to promote mammography participation for women with multiple chronic conditions.

## Introduction

Female breast cancer is the leading cause of global cancer incidence, with an estimated 2.3 million new cases, and the fifth leading cause of cancer mortality worldwide^1^. In 2020, the global cancer project (GLOBOCAN 2020) estimated increasing age-specific standardized incidence of 47.8 per 100,000 and mortality rates of 13.6 per 100,000^1^. In Taiwan, female breast cancer is also the leading cause of cancer. Age-standardized incidence rates increased from 28.4 per 100,000 in 1995 to 78.9 per 100,000 in 2018, and age-standardized mortality rates from 9.7 per 100,000 to 12.5 per 100,000^2^. To improve early diagnosis and survival outcomes, mammography has been suggested as an effective screening tool. The American Cancer Society recommends that average-risk women aged 45–54 years undergo mammography annually, and women aged 55 years or older biennially. A report from the 2013 National Health Interview Survey of America found that 69.1% of women aged 50 years or older are adherent to breast cancer screening guidelines every 2 years, while but mammography screening rates remain much lower in Asian countries^3^.

Chronic conditions and cancer may share common risk factors, including demographics (age, sex, ethnicity), and genetic and lifestyle-related factors (obesity, diet, physical activity, tobacco or alcohol consumption)^4–6^. Therefore, comorbidity substantially affects breast cancer risk and cancer stage at diagnosis^4,7–9^. However, existing studies suggest that women with chronic conditions are less likely to participate in breast cancer screening^10,11^. Similar findings were also found in one current study by Hsieh, which examined mammography participation among women aged 50–69 years at various health statuses in Taiwan and suggested chronically ill women tend to experience greater demand for medical visits and are willing to trade mammography screening for medical visits given the original budget and time constraints, and thus will have lower mammography uptake^11^.

Potential benefits of mammography uptake among women with different chronic conditions remain unclear. A systematic review by Braithwaite et al. included seven studies regarding the benefit or harm of mammography screening in women aged 65 years or older in relation to comorbidity, all conducted in the United States, and suggested that screening benefits may decrease with increasing age and comorbidity burden^12^. Demb et al. used mammography registry data to examine the effects of continuous mammography on risk of incident breast cancer and mortality among 222,088 screened women ages greater than 66 years old in the United States, and also found older women with increasing comorbidity may have diminished benefit from continuous screening^13^. Another recent study by Beau et al. used hospital electronic medical records in one screened and the other two non-screened control regions in Denmark to compare the effect of chronic diseases on risk of breast cancer mortality among women aged 50–69 years, and found marginal effect of mammography on breast cancer mortality in women with chronic diseases^14^. Neverthess, existing studies examining on benefits of mammography screening had mixed results and conducted in the United States or Europe^12–16^, very few conducted in Asia populations. In addition, there were still lack of nationwide population-based studies examining the magnitude of potential interaction effect between mammography uptake and chronic conditions on early detection or health outcomes.

The current study sought to use a population-based matched cohort study design to examine the potential benefit and moderation effect of mammography screening among women aged 50–69 years at various health statuses in Taiwan. Breast cancer screening policy in Taiwan is a key national cancer prevention policy. Since 2004, after passage of the National Cancer Prevention Law of 2003, Taiwan’s Ministry of Health Promotion Administration initiated an organized breast cancer screening strategy covering free biannual mammography services for the entire population of women aged 50–69 years^17^. Specifically, we identified entire female population aged 50–69 yeas in 2010 and used an exact matching approach to match women at different chronic illness levels. A generic comorbidity measure was used to characterize total chronic illness burden. We then compared the effect of mammographic screening on early diagnosis of breast cancer and all-cause mortality among matched cohorts at various health statuses.

## Methods

### Study design and data source

We used a matched cohort study design with at least 4-year follow-up using four nationwide population databases in Taiwan. The first was the National Health Insurance (NHI) administrative claims database, which includes more than 99% of Taiwan’s 23 million enrollees^18^. The NHI database provides information including enrollment status, comorbid conditions, preventive care use, and primary health providers for chronic conditions. The second database was a national breast cancer screening registry, 2004–2014, which collected accurate mammography dates for participants. The third was a national cancer registry, from which accurate diagnoses of overall cancer and breast cancer could be derived, 1979–2014^19,20^. The fourth was a national death registry, 2004–2014, which provides accurate death dates. We linked and analyzed these four population-based datasets with encrypted identifiers for the study population during 2020–2021 in the Health and Welfare Data Science Center of the Ministry of Health and Welfare, a government-operated national data warehouse.

### Ethical aspects

The study followed the ethical standards of the Institutional Review Board of the Kaohsiung Medical University Hospital (IRB number: KMUHIRB-E(I)-20190177) and the Helsinki Declaration of the World Medical Association. Consent to Participate: Given this research was retrospective study using secondary health administrative database, patients’ informed consent was waived.

### Study population

We first identified all women aged 50–69 years in 2010 (n = 2,564,252) eligible for free mammography screening in the national breast cancer screening program using the national NHI enrollment data. The study index date was defined as January 1, 2010, and the study end date as December 31, 2014. Each study cohort was followed from the index date to the first of study end date, or death. To avoid potential problems due to existing cancer disease or erroneous records, we excluded women with any record of cancer diagnosis (n = 143,619) or death (n = 487) before the index date. We then excluded women with any record of breast cancer screening services before the index date (n = 510,808) to ensure that all study subjects were new to mammography. To reduce potential bias due to missing data when measuring an individual’s health status, we further excluded women without medical records during follow-up (n = 33,536). A total of 1,867,802 women aged 50–69 years were included in this study.

**Figure 1:**
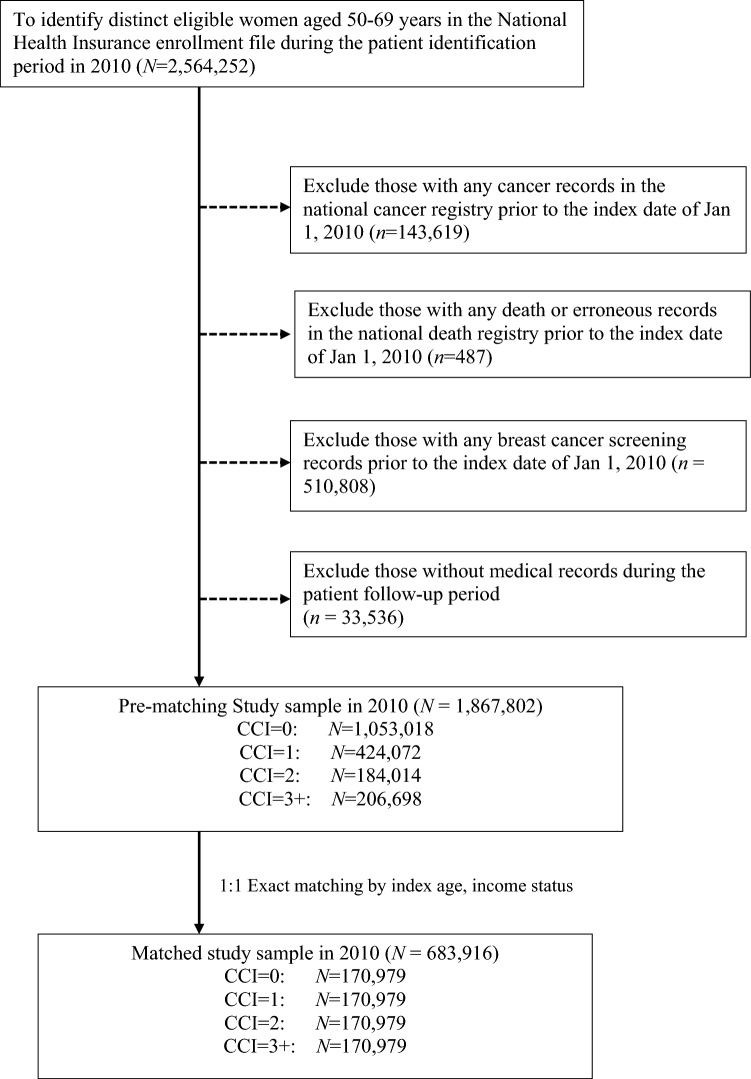
Inclusion and exclusion criteria in this study.

To identify levels of overall chronic illness severity in the study sample, we used the Deyo-Charlson Comorbidity Index (CCI), weighting comorbid conditions from the index date to the date of mammography, incident breast cancer, death, or study end date, whichever came first^21,22^. This index is an ICD-9-CM coding adaption and has been widely used by health researchers to measure general disease severity and case mix in health administrative claims databases, with low scores representing lowest risk^21,22^. The study sample was classified into four groups with CCI scores of 0, 1, 2, or 3+. As older age is concurrent with increased risk of chronic conditions and mortality, leading to selection bias and incomparable samples, we used a 1-to-1 exact matching approach to match groups with different overall chronic illness severity levels based on index age in years and baseline income status (< new Taiwanese dollar [NTD] 20,000, dependent, NTD 20,000–40,000, and NTD 40,001+) to create four pairs of exactly balanced groups^23^. Exact matching approach is one of matching methods frequently used in literature, which essentially matched each subgroups with exactly the same values on specific covariates and generated homogeneous comparable groups^23^. The sample sizes of each paired matched group with CCI scores of 0, 1, 2, or 3+ were 170,979. Figure 1 shows study inclusion and exclusion criteria.

### Measurements and variable definitions

#### Outcome of interest

We aimed to examine the effect of mammography on breast cancer diagnosis at early stage and all-cause mortality among women with various comorbid conditions. To measure all-cause mortality, we linked data with the National Death Registry and defined all-cause mortality as any death record after the index date. We then followed each subject until from the index date to the date of death, or study end date, whichever came first, and calculated total person-years for each subject for all-cause mortality. With respect to the measure of breast cancer diagnosis at early stage, we used the Taiwan cancer registry to identify incident breast cancer diagnosis after the index date using ICD-9-CM diagnosis code 174 or ICD-10-CM code C50. Based on the Tumor–Nodes–Metastasis staging system of the American Joint Committee on Cancer version 7 in the National Cancer Registry, breast cancer stages were categorized as 0–IV and early stage (0–II) or advanced stage (III, IV). In addition, we included a set of binary variables for mammography participation, and mammography access through in-reach in a hospital or out-reach in a community.

#### Other confounding baseline covariates

In addition to covariates such as the CCI and income status categories, we included two variables to identify health behavior characteristics (participation in any population-based pap smear cervical cancer screening program or adult physical examination program within the follow-up period). In Taiwan, the Health Promotion Administration, Ministry of Health and Welfare, provides government-initiated national population-based health promotion programs, including the two mentioned^24^. Women aged older than 30 years are eligible to participate in free annual pap smear screening under the national population-based cervical cancer screening program. Adults aged 40–65 years are eligible to participate in free physical examinations to receive routine blood tests and basic physical examination every 3 years, and those older than 65 years every year^24^. To address the issue of patients with multiple outpatient visits to different health care providers, we used a plurality provider algorithm to assign the most frequently visited hospitals or clinics based on administrative billing for the greatest numbers of care visits during follow-up^25^. Health care institution characteristics included accreditation level (medical center, regional hospital, local hospital, clinic), certification status for mammography screening (yes/no), and geographic location (Taipei, northern, central, southern, Kao-Ping, and eastern regions).

### Statistical analysis

Descriptive analyses using chi-square tests for categorical variables and t-tests for continuous variables. The Cochran–Armitage test for trend was used to test linear trends in frequencies of outcome measures among different chronic condition levels^26^. Individual-level conditional logistic regression models were used to compare outcome measures for mammography participation and detection breast cancer at early stage (0–II) and cox proportional hazards models for all-cause mortality among exactly matched women with different chronic conditions. Both statistical techniques were proper methods for matched data to address the sparse data problem and provide robust results^27^. Interaction terms for mammography and CCI were generalized to test the moderation effects of mammography on early breast cancer diagnosis and mortality^28^. If an effect exists, the moderating variable may change the direction or magnitude of the relationship between CCI and outcome measures and the interaction term will be statistically significant^28^. In addition, to contextualize the magnitude of the mammography moderation effect, we generated adjusted predicted probabilities (APPs) and marginal effects (MEs) of the probabilities^29^. These are used to quantify the average and incremental level of chronic illness associated with outcome measures^29,30^. Specifically, we generated APPs and MEs of each chronic illness severity level on early breast cancer diagnosis and mortality among women who did or did not undergo mammography. For each chronic illness level, we also generated MEs to compare the likelihood of severe chronic illness on outcome measures among women who did or did not undergo mammography. Data analysis was generated using SAS software, version 9.4 of the SAS Institute Inc., Cary, NC, USA. A p value < 0.05 was considered statistically significant.

## Results

Table 1 summaries baseline demographic, health behavior, and health care institution characteristics among women aged 50–69 years at different chronic illness levels in 2010. Before matching, cohorts with higher CCIs were older. Mean age among women with CCI 0, 1, 2, 3+ in 2010 was 56.25, 57.87, 58.87, and 60.38 years, respectively. After exact matching based on index age and income status, demographic characteristics among women at different CCI levels were comparable.

**Table 1:** Study cohort demographic and clinical characteristics and primary health care providers’ organizational characteristics among study women with different levels of chronic illness.

Variables	Pre-matching cohort					Matched cohort^a^				
	CCI = 0	CCI = 1	CCI = 2	CCI = 3+	p-value^b^	CCI = 0	CCI = 1	CCI = 2	CCI = 3+	p-value^b^
N	1,053,018	424,072	184,014	206,698		170,979	170,979	170,979	170,979	
**Women’ demographic characteristics**										
Age in years (Mean ± STD)^a^	56.25 (± 5.14)	57.87 (± 5.53)	58.87 (± 5.68)	60.38 (± 5.70)	< 0.001	59.36 (± 5.56)	59.36 (± 5.56)	59.36 (± 5.56)	59.36 (± 5.56)	1.000
Age categories (N, %)										
50–54	476,831 (45.28%)	142,370 (33.57%)	50,495 (27.44%)	40,133 (19.42%)	< 0.001	40,133 (23.47%)	40,133 (23.47%)	40,133 (23.47%)	40,133 (23.47%)	1.000
55–59	310,812 (29.52%)	125,694 (29.64%)	52,010 (28.26%)	52,004 (25.16%)		49,522 (28.96%)	49,522 (28.96%)	49,522 (28.96%)	49,522 (28.96%)	
60–64	168,161 (15.97%)	89,201 (21.03%)	43,237 (23.50%)	53,414 (25.84%)		43,052 (25.18%)	43,052 (25.18%)	43,052 (25.18%)	43,052 (25.18%)	
65–69	97,214 (9.23%)	66,807 (15.75%)	38,272 (20.80%)	61,147 (29.58%)		38,272 (22.38%)	38,272 (22.38%)	38,272 (22.38%)	38,272 (22.38%)	
Income status (N, %)^a,c^										
< NTD 20,000	150,843 (14.32%)	58,005 (13.68%)	26,069 (14.17%)	31,646 (15.31%)	< 0.001	25,205 (14.74%)	25,205 (14.74%)	25,205 (14.74%)	25,205 (14.74%)	1.000
Dependent	280,440 (26.63%)	132,443 (31.23%)	63,314 (34.41%)	82,820 (40.07%)		62,576 (36.60%)	62,576 (36.60%)	62,576 (36.60%)	62,576 (36.60%)	
NTD 20,000–40,000	468,303 (44.47%)	180,911 (42.66%)	74,892 (40.70%)	76,528 (37.02%)		67,897 (39.71%)	67,897 (39.71%)	67,897 (39.71%)	67,897 (39.71%)	
NTD 40,001+	153,432 (14.57%)	52,713 (12.43%)	19,739 (10.73%)	15,704 (7.60%)		15,301 (8.95%)	15,301 (8.95%)	15,301 (8.95%)	15,301 (8.95%)	
**Women’ health behavioral characteristics (N, %)**										
Receiving population-based pap smear screening within follow up period (N, %)										
No	448,375 (42.58%)	177,792 (41.92%)	79,555 (43.23%)	108,514 (52.50%)	< 0.001	78,619 (45.98%)	75,486 (44.15%)	75,122 (43.94%)	87,535 (51.20%)	< 0.001
Yes	604,643 (57.42%)	246,280 (58.08%)	104,459 (56.77%)	98,184 (47.50%)		92,360 (54.02%)	95,493 (55.85%)	95,857 (56.06%)	83,444 (48.80%)	
Receiving population-based adult physical examinations within follow up period (N, %)										
No	503,333 (47.80%)	166,320 (39.22%)	69,744 (37.90%)	88,006 (42.58%)	< 0.001	78,177 (45.72%)	66,243 (38.74%)	64,350 (37.64%)	73,608 (43.05%)	< 0.001
Yes	549,685 (52.20%)	257,752 (60.78%)	114,270 (62.10%)	118,692 (57.42%)		92,802 (54.28%)	104,736 (61.26%)	106,629 (62.36%)	97,371 (56.95%)	
**Primary health care providers’ characteristics**										
Accreditation level (N, %)										
Medical Center	105,313 (10.00%)	60,118 (14.18%)	30,565 (16.61%)	38,403 (18.58%)	< 0.001	17,997 (10.53%)	24,519 (14.34%)	28,264 (16.53%)	31,687 (18.53%)	< 0.001
Regional Hospital	134,006 (12.73%)	71,877 (16.95%)	35,982 (19.55%)	48,697 (23.56%)		22,169 (12.97%)	29,101 (17.02%)	33,523 (19.61%)	40,144 (23.48%)	
Local Hospital	95,131 (9.03%)	47,445 (11.19%)	22,546 (12.25%)	29,415 (14.23%)		15,974 (9.34%)	19,405 (11.35%)	21,014 (12.29%)	24,152 (14.13%)	
Clinics	718,568 (68.24%)	244,632 (57.69%)	94,921 (51.58%)	90,183 (43.63%)		114,839 (67.17%)	97,954 (57.29%)	88,178 (51.57%)	74,996 (43.86%)	
Mammography certification (N, %)										
No	780,993 (74.17%)	277,076 (65.34%)	110,768 (60.20%)	111,189 (53.79%)	< 0.001	125,451 (73.37%)	111,359 (65.13%)	103,045 (60.27%)	92,184 (53.92%)	< 0.001
Yes	272,025 (25.83%)	146,996 (34.66%)	73,246 (39.80%)	95,509 (46.21%)		45,528 (26.63%)	59,620 (34.87%)	67,934 (39.73%)	78,795 (46.08%)	
NHI branch (N, %)										
Taipei	371,797 (35.31%)	148,509 (35.02%)	62,989 (34.23%)	64,526 (31.22%)	< 0.001	59,305 (34.69%)	59,623 (34.87%)	58,171 (34.02%)	53,419 (31.24%)	1.000
Northern	146,004 (13.87%)	58,190 (13.72%)	24,402 (13.26%)	27,358 (13.24%)		23,801 (13.92%)	23,336 (13.65%)	22,828 (13.35%)	22,401 (13.10%)	
Central	191,240 (18.16%)	74,823 (17.64%)	32,048 (17.42%)	38,248 (18.50%)		30,933 (18.09%)	30,583 (17.89%)	29,905 (17.49%)	31,599 (18.48%)	
Southern	138,465 (13.15%)	60,606 (14.29%)	28,523 (15.50%)	32,241 (15.60%)		22,928 (13.41%)	24,322 (14.23%)	26,469 (15.48%)	26,695 (15.61%)	
Kao-Ping	184,951 (17.56%)	71,427 (16.84%)	30,499 (16.57%)	37,690 (18.23%)		30,641 (17.92%)	28,985 (16.95%)	28,454 (16.64%)	31,432 (18.38%)	
Eastern	20,561 (1.95%)	10,517 (2.48%)	5553 (3.02%)	6635 (3.21%)		3371 (1.97%)	4130 (2.42%)	5152 (3.01%)	5433 (3.18%)	

*CCI* modified Charlson comorbidity index, *NHI* National Health Insurance Administration, *NTD* New Taiwanese Dollar.

^a^Exact matching approach was used to 1-to-1 match groups of samples with different level of overall chronic illness severity based on their index age in years and baseline income status.

^b^p-value for comparison of patients’ and health care providers’ characteristics between screened and non-screened women; chi-square tests were used for categorical variables and t-tests for continuous variables.

^c^Income status was presented in New Taiwanese Dollar (NTD). The exchange rate between NTD and US Dollar is about 30:1 in this study.

Table 2 compares mammography participation, newly diagnosed breast cancer stages, and all-cause mortality among women at different levels of chronical illness severity. The Cochran–Armitage test for trend was used to compare frequencies of outcome measures across chronic illness levels. Mammography rates and repeat participation rates decreased as chronic illness levels increased (p < 0.001). Approximately 0.92% to 1.25% of matched cohorts at each CCI level were newly diagnosed with breast cancer. The proportion of early breast cancer diagnosis decreased as the chronic illness level increased (p < 0.001), while the all-cause mortality rate increased (p < 0.001).

**Table 2:** Associations between different levels of chronic illness and outcomes of interest among matched cohorts.

Variables	Matched cohort				
	CCI = 0	CCI = 1	CCI = 2	CCI = 3+	p-value^a^
N	170,979	170,979	170,979	170,979	
**Mammography utilization**					
Mammography participation during follow-up period (N, %)					
No	90,925 (53.18%)	99,169 (58.00%)	107,156 (62.67%)	125,604 (73.46%)	< 0.001
Yes	80,054 (46.82%)	71,810 (42.00%)	63,823 (37.33%)	45,375 (26.54%)	
Repeated mammography during follow-up period (N, %)					
No repeated mammography	138,191 (80.82%)	145,830 (85.29%)	151,285 (88.48%)	159,979 (93.57%)	< 0.001
Repeated mammography (at least twice during the observation period)	32,788 (19.18%)	25,149 (14.71%)	19,694 (11.52%)	11,000 (6.43%)	
Repeated mammography during follow-up period among those received mammography (N, %)					
No repeated mammography	47,266 (59.04%)	46,661 (64.98%)	44,129 (69.14%)	34,375 (75.76%)	< 0.001
Repeated mammography (at least twice during the observation period)	32,788 (40.96%)	25,149 (35.02%)	19,694 (30.86%)	11,000 (24.24%)	
Assess of mammography services among those received mammography (N, %)					
Inreach (through in-hospital examination)	43,739 (54.64%)	41,720 (58.10%)	38,861 (60.89%)	28,355 (62.49%)	< 0.001
Outreach (through mammography car)	36,315 (45.36%)	30,090 (41.90%)	24,962 (39.11%)	17,020 (37.51%)	
**Breast cancer diagnoses**					
Newly diagnosed as breast cancer (N, %)					
No	168,834 (98.75%)	169,301 (99.02%)	169,211 (98.97%)	169,404 (99.08%)	< 0.001
Yes	2145 (1.25%)	1678 (0.98%)	1768 (1.03%)	1575 (0.92%)	
Breast cancer stage (N, %)^c^					
Stage 0	284 (13.24%)	267 (15.91%)	235 (13.29%)	152 (9.65%)	< 0.001
Stage I	650 (30.30%)	562 (33.49%)	511 (28.90%)	394 (25.02%)	
Stage II	688 (32.07%)	514 (30.63%)	569 (32.18%)	432 (27.43%)	
Stage III	284 (13.24%)	188 (11.20%)	251 (14.20%)	227 (14.41%)	
Stage IV	136 (6.34%)	80 (4.77%)	110 (6.22%)	278 (17.65%)	
Missing values/ or misclassification^b^	103 (4.80%)	67 (3.99%)	92 (5.20%)	92 (5.84%)	
Breast cancer early/late stage (exclude missing) during follow-up period (N, %)^c^					
Early stage (0,1,2)	1622 (79.43%)	1343 (83.36%)	1315 (78.46%)	978 (65.95%)	< 0.001
Late stage (3,4)	420 (20.57%)	268 (16.64%)	361 (21.54%)	505 (34.05%)	
All-cause mortality (N, %)					
No	168,979 (98.83%)	168,028 (98.27%)	165,805 (96.97%)	155,997 (91.24%)	< 0.001
Yes	2000 (1.17%)	2951 (1.73%)	5174 (3.03%)	14,982 (8.76%)	
Total person-years of follow-up (mean ± STD)	4.97 (± 0.26)	4.96 (± 0.32)	4.93 (± 0.42)	4.82 (± 0.67)	< 0.001

*CCI* modified Charlson comorbidity index.

^a^p-value was generated by using Cochran-Armitage tests for trend.

^b^Misclassification were those TNM classifications in the national cancer registry, which were not missing, but with codes like “999/99” (unclear or physicians did not code), “888/88” (non-applicable) , “BBB/BB/B” or “X” (TX, NX, MX, occult carcinoma, found cancer cell but may not a specific tumor).

^c^Only those with newly diagnosed as breast cancer were analyzed.

Table 3 provides results from conditional logistic regression models and cox proportional hazards models, which examined the effects of chronic illness on mammography participation (Model 1); and early diagnosis of breast cancer (Model 2); the interaction of CCI and mammography on early diagnosis of breast cancer (Model 3); all-cause mortality (Model 4); and the interaction effect on all-cause mortality (Model 5). Compared with CCI score 0, women with more severe chronic conditions were less likely to participate in mammography screening, less likely to be diagnosed at an early stage of breast cancer, and at higher risk of all-cause mortality. Mammography participation increased the likelihood of early breast cancer diagnosis (OR 1.48, 95% CI 1.36–1.60) and decreased risk of all-cause mortality (HR 0.53, 95% CI 0.51–0.55). The interaction terms of CCI and mammography participation indicated statistically significantly increased benefits of early breast cancer diagnosis and decreased risk of all-cause mortality as chronic illness increased. With respect to other covariates related to health behaviors, women received population-based pap smear screening were more likely participate in mammography (OR 11.05, 95% CI 10.87, 11.25), be diagnosed at early stage of breast cancer (OR 2.22, 95% CI 2.04, 2.41) and lower risk of all-cause mortality (HR 0.56, 95% CI 0.55, 0.58). Women received population-based physical examination were more likely participate in mammography (OR 1.80, 95% CI 1.78, 1.83), and lower risk of all-cause mortality (HR 0.51, 95% CI 0.49, 0.52).

**Table 3:** Study results of mammography uptake and detection of breast cancer at early stage using conditional logistic regression models and all-cause mortality prevention using cox proportional hazard models among women with different levels of chronic illness.

Covariates/models	Model 1^a^: mammography participation		Model 2^a^: breast cancer diagnosis at early stage (0,1,2)		Model 3^a^: breast cancer diagnosis at early stage (0,1,2) (Interaction)		Model 4: all-cause mortality^b^		Model 5: all-cause mortality (Interaction)^b^	
	OR (95% CI)	p-value	OR (95% CI)	p-value	OR (95% CI)	p-value	HR (95% CI)	p-value	HR (95% CI)	p-value
**Mammography participation (ref. = no)**										
Yes			1.48 (1.36, 1.60)	< 0.001	1.03 (0.90,1.18)	0.672	0.53 (0.51, 0.55)	< 0.001	0.91 (0.83, 1.00)	0.058
**Women’ chronic illness characteristics**										
CCI categories (Ref.: CCI = 0)										
1	0.66 (0.65, 0.67)	< 0.001	0.80 (0.74, 0.87)	< 0.001	0.61 (0.53, 0.69)	< 0.001	1.44 (1.36, 1.52)	< 0.001	1.56 (1.46, 1.68)	< 0.001
2	0.48 (0.47, 0.49)	< 0.001	0.79 (0.73, 0.85)	< 0.001	0.66 (0.58, 0.75)	< 0.001	2.41 (2.29, 2.54)	< 0.001	2.77 (2.60, 2.94)	< 0.001
3+	0.29 (0.29, 0.30)	< 0.001	0.60 (0.55, 0.65)	< 0.001	0.41 (0.36, 0.47)	< 0.001	5.97 (5.69, 6.26)	< 0.001	7.16 (6.76, 7.58)	< 0.001
Interaction of mammography participation and levels of chronic illness burden										
CCI: 1 × mammography participation					1.62 (1.34, 1.96)	< .0001			0.77 (0.68, 0.87)	< 0.001
CCI: 2 × mammography participation					1.35 (1.12, 1.62)	0.002			0.63 (0.56, 0.70)	< 0.001
CCI: 3 + × mammography participation					2.08 (1.70, 2.54)	< 0.001			0.45 (0.40, 0.50)	< 0.001
**Women’ health behavioral characteristics**										
Receiving population-based pap smear screening within follow up period (Ref.: no)										
Yes	11.05 (10.87, 11.25)	< 0.001	2.22 (2.04, 2.41)	< 0.001	2.24 (2.06, 2.44)	< 0.001	0.56 (0.55, 0.58)	< 0.001	0.56 (0.54, 0.58)	< 0.001
Receiving population-based adult physical examinations within follow up period (Ref.: no)										
Yes	1.80 (1.78, 1.83)	< 0.001	0.88 (0.82, 0.95)	0.001	0.89 (0.83, 0.96)	0.003	0.51 (0.49, 0.52)	< 0.001	0.50 (0.49, 0.52)	< 0.001
**Primary health care providers’ characteristics**										
Accreditation level (Ref.: Medical Center)										
Regional Hospital	1.10 (1.08, 1.13)	< 0.001	0.72 (0.66, 0.80)	< 0.001	0.72 (0.65, 0.79)	< 0.001	0.89 (0.86, 0.92)	< 0.001	0.89 (0.86, 0.92)	< 0.001
Local Hospital	1.04 (1.01, 1.08)	0.011	0.40 (0.35, 0.47)	< 0.001	0.40 (0.35, 0.46)	< 0.001	0.73 (0.70, 0.77)	< 0.001	0.73 (0.70, 0.77)	< 0.001
Clinics	0.86 (0.83, 0.89)	< 0.001	0.32 (0.27, 0.38)	< 0.001	0.32 (0.27, 0.38)	< 0.001	0.43 (0.41, 0.45)	< 0.001	0.43 (0.41, 0.46)	< 0.001
Mammography certification (Ref.: no)										
Yes	1.59 (1.54, 1.64)	< 0.001	1.44 (1.25, 1.66)	< 0.001	1.44 (1.25, 1.66)	< 0.001	0.92 (0.88, 0.97)	< 0.001	0.92 (0.88, 0.97)	< 0.001
NHI branch (Ref.: Taipei)										
Northern	1.12 (1.09, 1.15)	< 0.001	0.89 (0.79, 0.99)	0.040	0.89 (0.79,1.00)	0.043	1.40 (1.35, 1.46)	< 0.001	1.40 (1.34, 1.46)	< 0.001
Central	0.99 (0.97, 1.02)	0.613	0.94 (0.85, 1.04)	0.214	0.94 (0.85,1.04)	0.243	1.43 (1.38, 1.48)	< 0.001	1.43 (1.37, 1.48)	< 0.001
Southern	1.17 (1.14, 1.20)	< 0.001	0.97 (0.87, 1.09)	0.609	0.97 (0.87,1.09)	0.595	1.58 (1.52, 1.65)	< 0.001	1.58 (1.52, 1.64)	< 0.001
Kao-Ping	1.09 (1.07, 1.11)	< 0.001	0.98 (0.88, 1.08)	0.678	0.98 (0.88,1.08)	0.676	1.55 (1.50, 1.61)	< 0.001	1.55 (1.50, 1.61)	< 0.001
Eastern	1.17 (1.12, 1.22)	< 0.001	0.88 (0.70, 1.10)	0.250	0.88 (0.70,1.10)	0.267	1.85 (1.72, 1.98)	< 0.001	1.85 (1.72, 1.98)	< 0.001

*Ref.* reference group, *OR* odds ratio, *HR* hazard ratio, *CI* confidence interval, *NHI* National Health Insurance Administration, *CCI* modified Charlson comorbidity index.

^a^Multivariable conditional logistic regression model was analyzed.

^b^Multivariable cox proportional hazard model was analyzed.

Table 4 shows results of mean APPs for the probability of early breast cancer diagnosis and all-cause mortality among different chronic illness levels, and MEs of predicted probability between women who did and did not undergo mammography. The APPs of early diagnosis decreased for CCI scores 0, 1, 2, 3+ among both women with and without participating in mammography screening. Positive MEs of early breast cancer diagnosis between women who did and did not participate indicated the magnitude of benefit of mammography across chronic illness levels. The APPs of all-cause mortality increased for CCI scores 0, 1, 2, 3+ among women with and without participating in mammography screening. The MEs of all-cause mortality between women who did and did not participate at each chronic severity level indicated that mammography reduced the risk of mortality as chronic illness severity levels increased.

**Table 4:** Results of adjusted predicted probabilities and marginal effects of the levels of chronic illness and probabilities of outcome of interests among matched cohorts with and without mammography participation.

Outcomes	Mammography Participation (Yes)	Mammography Participation (No)	ME of chronical illness among women with mammography (yes)	ME of chronical illness among women without mammography (no)	ME of mammography by different levels of chronic illness (Ref.: with mammography participation versus without)
	APP (%, 95% CI)	APP (%, 95% CI)			
**Early diagnosis of breast cancer**					
CCI					
0	1.23 (1.17, 1.30)	0.88 (0.82, 0.93)	Ref.	Ref.	0.35 (0.29, 0.42)
1	0.92 (0.87, 0.98)	0.65 (0.61, 0.70)	− 0.31 (− 0.39, − 0.23)	− 0.22 (− 0.28, − 0.17)	0.27 (0.21, 0.32)
2	0.86 (0.80, 0.91)	0.61 (0.57, 0.65)	− 0.37 (− 0.45, − 0.30)	− 0.27 (− 0.32, − 0.21)	0.25 (0.20, 0.30)
3+	0.65 (0.60, 0.70)	0.46 (0.43, 0.49)	− 0.58 (− 0.65, − 0.51)	− 0.41 (− 0.47, − 0.36)	0.19 (0.15, 0.23)
**All-cause mortality**					
CCI					
0	0.81 (0.77, 0.85)	1.52 (1.45, 1.59)	Ref.	Ref.	− 0.71 (− 0.76, − 0.66)
1	1.16 (1.10, 1.21)	2.17 (2.09, 2.24)	0.35 (0.29, 0.41)	0.65 (0.55, 0.75)	− 1.01 (− 1.07, − 0.94)
2	1.93 (1.85, 2.01)	3.57 (3.48, 3.67)	1.12 (1.05, 1.19)	2.06 (1.94, 2.17)	− 1.64 (− 1.74, − 1.55)
3+	4.77 (4.61, 4.94)	8.55 (8.41, 8.70)	3.97 (3.82, 4.12)	7.04 (6.88, 7.19)	− 3.78 (− 3.98, − 3.58)

*CCI* modified Charlson comorbidity index, *Ref.* reference group, *ME* marginal effects, *APP* adjusted predicted probabilities.

## Discussions

This study examined potential benefits and moderation effects of mammography screening on early breast cancer diagnosis and mortality among women aged 50–69 years at various health statuses in Taiwan. A generic CCI measure was used to identify women at different levels of overall chronic illness burden. Consistent with previous literature, our findings indicate that women at higher chronic illness levels were less likely to participate in mammography screening and to have breast cancer newly diagnosed at early stages, and were at greater risk of all-cause mortality^4,7–9^. Our findings further provide empirical evidence that mammography may moderate the association between chronic illness burden and early breast cancer diagnosis and mortality.

Comorbidity may present barriers to breast cancer screening and complicate diagnostic decision-making^7,10^, and may substantially affects medical prognosis^4,7–9^. Similar to findings from a systematic review and meta-analysis study based on few high-quality studies in Europe or in the United States by Diaz et al. and a recently study conducted in Taiwan by Hsieh^10,11^, our study findings indicate that women aged 50–69 years at higher chronic illness levels were less likely to participate in mammography screening in Taiwan. As Fleming et al. indicated, comorbidity as a predictor of newly diagnosed breast cancer stage, which may relate to several hypotheses: the interaction between comorbid conditions and cancers at the cellular level may increase risks of metastasis, or comorbid conditions may constitute a competing demand against use of preventive services. Additionally, as observed clinically, physicians are more likely to request mammography for women at higher general risk of breast cancer (e.g., family history), but less likely to request it for average-risk women due to greater levels of chronic illnesses^7^. These all may lead to lower mammography utilization and thus exacerbate the odds of late-stage breast cancer diagnosis and prognosis among women with chronic illness.

To the best of our knowledge, this study is the first to use entire population data to examine the potential benefits of mammography screening on early breast cancer diagnosis and all-cause mortality among women with different chronic conditions in Asian countries. Most existing literature conducted in European countries or in the United States, and found mixed results^12–16^. In general, our findings supported the benefit of mammography screening among women with multiple chronic conditions, which increased the likelihood of early stage breast cancer diagnosis and decreased odds of all-cause mortality. Specifically, mammography screening significantly moderated the link between chronic illness burden and late-stage diagnosis and risk of all-cause mortality. The presence of chronic diseases is an important factors to consider in organized population-based mammography screening program among women with chronic conditions to improve their potential benefits and health outcomes from screening.

Our study has several strengths. It used four longitudinal nationwide population-based datasets linking NHI administrative claims, national cancer registry, death registry, and breast cancer screening registry in Taiwan. These included all women aged 50–69 years in 2010, or approximately 2.5 million population, and provided accurate screening attendance information for identifying screened and non-screened groups. In addition, the study database allowed us to generate a generic composite measure of total chronic illness burden from NHI administrative data, reducing potential recall bias from self-reported health status^10^, or missing information due to using data from regional hospital-based electronic medical records^14^. In addition, Czwikla et al. addressed methodology concern of selection bias issue as the results of mammography screening participants and nonparticipants are not comparable regarding various health statuses^31^. To avoid potential selection bias as Czwikla et al. point out, we used an exact matching approach to generate balanced groups with different burdens of chronic conditions based on birth years and income status and compared outcomes of interest at the same chronic condition levels between screened and non-screened groups.

Nevertheless, this study also has several limitations. First, to compare results with existing studies^13,14^, we used the CCI to measure overall burden of chronic conditions. Future studies may use other types of comorbidity measures to investigate benefits of mammography among women at different health statuses. Second, given the study data primarily was derived from health insurance administrative claim database, some unobservable confounders are unavailable when investigating research questions, such as education level, body weight, lifestyle factors and habits or breast cancer awareness. Some cautions were raised when interpreting for the medication effect of mammography and comorbid conditions on all-cause mortality. Finally, the data were from women aged 50–69 years in Taiwan. Results may not generalize to other health systems in other countries.

In conclusion, analyzing national population-based data in Taiwan, this study provides empirical evidence with respect to the moderation effect of mammography screening, which increased likelihood of early stage breast cancer diagnosis and decreased odds of all-cause mortality. Public health policy and strategies may be necessary to improve mammography participation and early detection efforts for women with chronic conditions.
